# Comprehensive Analysis of Cellular Senescence-Related Genes in Prognosis, Molecular Characterization and Immunotherapy of Hepatocellular Carcinoma

**DOI:** 10.1186/s12575-022-00187-7

**Published:** 2022-12-19

**Authors:** Liang Sun, Zitao Liu, Ke Ning, Zhipeng Wu, Zhendong Chen, Zhengyi Wu, Xiangbao Yin

**Affiliations:** 1 Department of General Surgery, The Second Affiliated Hospital of Nanchang University, Nanchang, China; 2 Department of Emergency, The First Affiliated Hospital of Nanchang University, Nanchang, China

**Keywords:** Cellular senescence, Prognosis, Signature, Immunotherapy, Hepatocellular carcinoma

## Abstract

**Background:**

Cellular senescence is a tumor suppressive response in which the cell cycle is in a state of permanent arrest and can inhibit tumor cell proliferation. In recent years, induction of cellular senescence has been shown to be important for antitumor therapy, and the link between cellular senescence and clinical prognosis and immunotherapy of hepatocellular carcinoma is still unknown.

**Methods:**

We performed enrichment analysis of genes in three cellular senescence gene sets, screened for gene sets significantly enriched in hepatocellular carcinoma and extracted genes from them. Signature were constructed using senescence-related genes, and their expression was verified at the protein and RNA levels. Survival, clinical staging and grading, immune infiltration, immunotherapy, and drug sensitivity were also analyzed between risk groups.

**Results:**

The q-PCR and immunohistochemistry results revealed significant differences in the expression of the signature genes between normal and tumor tissues. Significant differences in clinicopathological features, prognosis and immune infiltration were observed between risk groups. In the low-risk group, better OS and lower TMB scores were demonstrated, while the high-risk group had higher immune checkpoint expression, as well as lower risk of immune escape. In addition, we found that the High-risk group was more sensitive to sorafenib.

**Conclusion:**

In summary, the signature constructed using aging-related genes can reliably predict patient prognosis and immunotherapy efficacy, providing a new idea for immune system therapy of hepatocellular carcinoma.

**Supplementary Information:**

The online version contains supplementary material available at 10.1186/s12575-022-00187-7.

## Introduction

Hepatocellular carcinoma (HCC) is currently the sixth most common tumor worldwide, accounting for approximately 5% of all cancers [1]. It has the fourth highest mortality rate, with approximately 745,000 deaths from hepatocellular carcinoma each year [2]. There is no good treatment for HCC, and although its treatment options include: surgery, interventional and molecular targeted therapy, the mortality rate of HCC has not been effectively controlled [3].

Cellular senescence is a marker of biological and temporal aging, and a potential indicator of pathological tissue status [4]. Cellular senescence refers to the state in which cells reach cycle arrest and is also aimed at cell clearance or cancer prevention. In contrast to apoptosis, senescence-defined living cells are able to communicate with neighboring cells and the immune system by secreting powerful extracellular factors [5, 6]. Senescent cells are involved in immune system clearance and tissue repair, and senescence appears to be more powerful compared to apoptosis, which has only a transient signaling capacity [7].

Cellular senescence is considered to be the response of proliferating somatic cells to exogenous and endogenous stress and injury. It is characterized by a permanent blockage of the cell cycle [8]. Cellular aging is inevitable over time and is accompanied by the degradation of many physiological functions, making it a detrimental factor in many diseases of the body [9]. Cellular senescence has now been found to be an important way to control tumor progression and inhibit the proliferation of cancer cells by inducing senescence [7, 10]. Many therapeutic agents induce cellular senescence, called therapeutically induced senescence (TIS), which can effectively inhibit tumor development [11, 12]. However, it has also been found that if senescent cells persist, they may also contribute to the development of tumors [13, 14]. Therefore, it is crucial how to effectively use senescence-inducing drugs to inhibit tumor development. With the discovery of senescence-associated secretory phenotypes (SASP), the long-term and long-lasting effects of senescent cells on the balance of the tissue internal environment are also gaining attention [15]. In recent years, studies on senescence-associated genes as diagnostic and prognostic markers for tumors have begun to emerge, but no studies have been reported on the use of senescence-associated genes to predict prognosis and immunotherapy for HCC.

Therefore, in this study, we constructed a signature for predicting HCC prognosis by targeting cellular senescence-related genes, providing new insights into the prognosis and immunotherapeutic targets of HCC.

## Materials and Methods

### Data Processing

First, we entered the Gene Set Enrichment Analysis (GSEA) database, then entered the MsigDB section and click Search, and search for cellular senescence gene sets. We screened three gene sets: “GOBP_CELL_AGING”, “GOBP_REGULATION_OF_CELL_AGING” and “REACTOME_CELLULAR_SENESCENCE”.

342 HCC patients (survival time > 31 days) were obtained from the TCGA database. 231 HCC patients were obtained from the ICGC database (ICGC-LIRI-JP) (metastatic hepatocellular carcinoma and patients with missing data were excluded). Variance analysis was performed using the "limma" R package, with FDR values set to less than 0.05 and logFCfilter set to greater than 0.5.

### Screening of Senescence-Related Genes

Screening of senescence-related genes using Weighted Gene Co-expression Network Analysis (WGCNA) algorithm. The minimum number of module genes was set to 30, and the gene hierarchy clustering dendrogram was pruned using the shear dynamic function to generate co-expression modules. The differences in module signature genes (ME) were calculated using the module signature gene function, and the modules with the highest correlation coefficients were extracted for further study.

### Development and Validation of Senescence-Related Genes

The differential expressed genes (DEGs), prognostic genes and the highest correlated modular genes from WGCNA in TCGA were taken as the intersection set, and the obtained genes were used for signature construction. The 342 HCC patients were divided into a training set (set1) and a Test set (set2) (1:1 randomized assignment), with the overall TCGA dataset as the validation set (set3) and external validation using the ICGC dataset (set4). Risk signature were constructed from the training set (Lasso and COX regression analysis)(RiskScore = expression level of mRNA ∗ The regression coefficient).

The validation groups (set 2, 3 and 4) were divided into high and low risk groups, followed by survival analysis and plotting of ROC curves (1, 2, and 3 years). The age, sex, Stage staging, A nomogram based on senescence-related genetic features was constructed using "rms" and "regplot" (R package) to combine rank staging and risk scores. The accuracy and reliability of the nomogram is judged from the calibration curve. Subsequently, the signature was further validated in three validation cohorts (set2, set3 and set4). In addition, a comparative analysis with other HCC signatures was performed to further determine the accuracy of our signature [16–19].

### Expression Validation of Senescence-Related Signature Genes

After obtaining informed consent from patients, we collected 30 pairs of HCC tissues and paraneoplastic tissues (from the Second Affiliated Hospital of Nanchang University), while one normal hepatocyte line (7702) and four HCC cell lines (97H, LM3, HepG2 and 7721) were cultured (cells were from the Shanghai Institute of Cell Biology). All cell lines were cultured in high glucose DMEM (Solarbio, Beijing, China) supplemented with 10% fetal bovine serum (bio-Industries, Beit-Haemek, Israel), 100 µg/ml streptomycin and 100 U/mL penicillin at 37 °C, and in a 5% CO2 humidified incubator.

#### RNA Levels to Detect Expression Differences

Tissues and cells were extracted for total RNA. total RNA was extracted according to the instructions of Trizol kit (Invitrogen). cDNA was synthesized using reverse transcription kit (Takara). qRT-PCR was used to detect mRNA expression levels of the characterized genes. The primer sequences of the signature genes are shown in Table 1.

**Table 1:** Primer sequences used for RT-qPCR

Gene	Sequence (5’-3’)
GAPDH	F: GGAGCGAGATCCCTCCAAAAT
	R: GCTGTTGTCATACTTCTCATGG
CBX2	F: GACTTAGATGCTAAGAGGGGTC
	R: CTTCTTCCGGATGGGATCCTTC
CDKN2B	F: CAGCGATGAGGGTCTGGC
	R: CCTCCCGAAACGGTTGACTC
ETS2	F: CTCTGGGCCACCAATGAGTT
	R: TCACCCACAAAGTCAGGTGC
HMGA1	F: CAGCGAAGTGCCAACACCTA
	R: GTCTGCCCCTTGGTTTCCTT
UBE2S	F: GATCTTCCACCCGAACGTGG
	R: CTCGTTGAGTGCAGACTCGG

#### Western Blot was Used to Detect Protein Expression Differences

HCC cell lines and tissue samples were extracted with RIPA lysate for total protein, and protein concentrations were determined by the BCA method. Each group of proteins was sampled and subjected to SDS-PAGE electrophoresis, electrotransferred to PVDF membrane, closed with 5% skimmed milk for 2 h, incubated with primary antibody overnight at 4 °C, washed 3 times with TBST for 10 min each time, and then the corresponding secondary antibody was added and incubated for 2 h at room temperature, washed 3 times with TBST for 10 min each time for fluorescent color development.

#### Immunohistochemistry (IHC) Experiments was Used to Detect Protein Expression Differences

HCC tissues were paraffin-embedded, sectioned, dewaxed and hydrated, incubated with anti-trait gene antibodies overnight at room temperature, then labeled with secondary antibodies for 30 min, stained and photographed.

The specific primary antibodies were purchased from the following resource:

GAPDH(Abmart, M20006, WB(1:5000)), CBX2(Abmart, PH3521, WB(1:1000), IHC(1:100)), CDKN2B(Affbiotech, AF0230, WB(1:1000), IHC(1:100)), ETS2(Abmart, MG225391, WB(1:1000), IHC(1:150)), HMGA1(ABclonal, A4343, WB(1:1000), IHC(1:100)), UBE2S(Abmart, PK66136, WB(1:1000), IHC(1:100)).

### Immune Cell Infiltration Analysis

The content of 22 human immune cell subpopulations in TCGA-LIHC was assessed using the CIBERSORT algorithm, followed by visual analysis of differences in immune cells between risk groups.

### Analysis of Tumor Microenvironment

In order to further verify the relationship between the constructed signature and tumor microenvironment and immunotherapy, the "ggplot" R package was used to analyze the degree of Tumor mutational burden (TMB) and Microsatellite Instability (MSI) among different risk groups. Then, the samples were divided into high TMB(H-TMB) and low TMB(L-TMB) according to the median value of TMB to compare whether there was difference in survival between the two groups. At the same time, in order to further reflect the survival difference between high and low risk groups, we also analyzed the survival difference between high TMB and low TMB among different risk groups.

### Immunotherapy and Drug Sensitivity Analysis

The immune escape relationship was compared between the risk groups by the TIDE algorithm. In addition, the difference in IC50 of sorafenib between the two groups and the correlation with the risk score were compared using the "pRRophetic" (R package).

### Statistical Analysis

The R language (version 4.1.2) and GraphPad Prism 8.0 were used for statistical analysis. the Chi-square test was used for correlation analysis of categorical data, *P* value < 0.05 indicates statistical significance.

## Results

### The Senescence Process of Cells was Activated Significantly in Hepatocellular Carcinoma

**Fig. 1:**
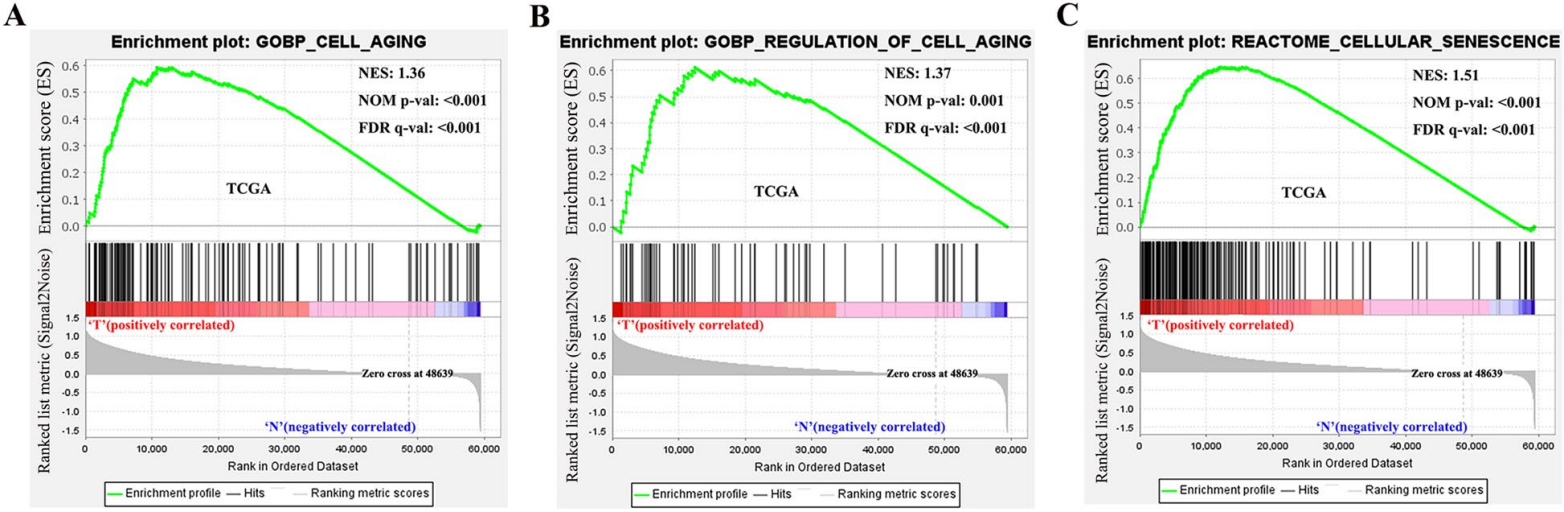
Gene Set Enrichment Analysis (GSEA). Three senescence-related gene sets were significantly activated in HCC tissues compared with normal tissues. The significance criteria were nominal *P*-value < 0.05 and FDR *q*-value < 0.25

Based on TCGA dataset, we performed enrichment analysis of "GOBP_CELL_AGING", "GOBP_REGULATION_OF_CELL_AGING" and "REACTOME_CELLULAR_SENESCENCE" and found that senescence-related genomes were significantly activated in HCC (Table S1) (Fig. 1A-C).

### Differential Expression and Prognostic of Senescence-Related Genes in TCGA-LIHC

**Fig. 2:**
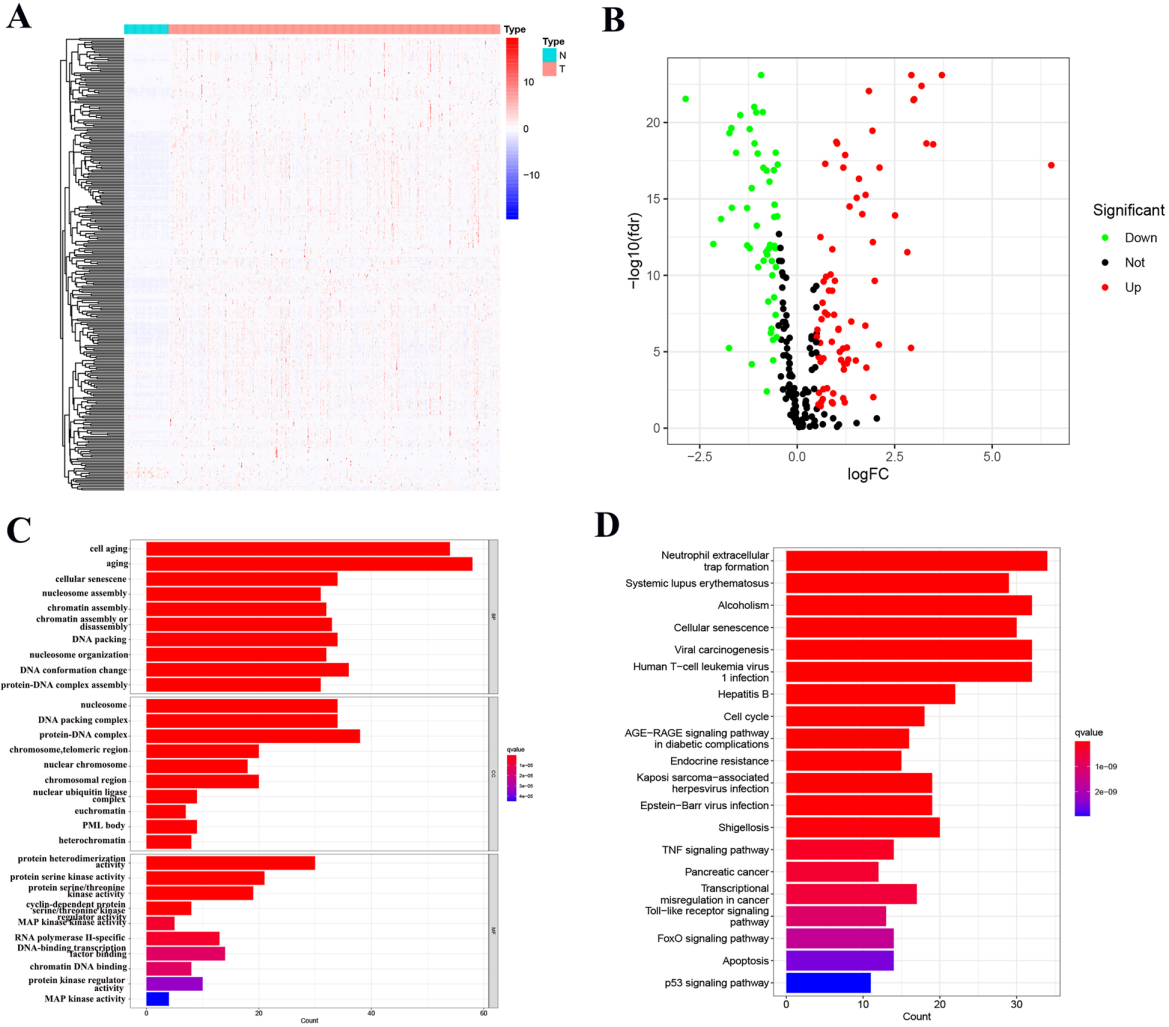
Identification of differentially expressed genes (DEGs) in HCC. **A** The heatmap. **B** The volcano map. **C** Gene ontology (GO) analysis of DEGs. **D** Kyoto Encylopedia of Genes and Genomes (KEGG) analysis of DEGs

After the three gene sets were opened in text form, the genes in the gene set were extracted, and we got 299 genes after removing duplicate genes. Then, we extracted these genes from TCGA-LIHC for differential expression analysis, and obtained 126 genes with significant differences (52 down-regulated and 74 up-regulated) (Fig. 2A, B) (Table S2). We subjected the differential genes to GO and KEGG enrichment analysis, and the results of GO enrichment analysis showed that they were mainly associated with cell aging, aging, cellular senescence, nucleosome and protein-DNA complex. KEGG enrichment analysis showed that it was mainly related to Neutrophil extracellular trap formation, Systemic lupus erythematosus, Alcoholism, Cellular senescence and Viral carcinogenesis (Fig. 2C, D).

In addition, after screening the information of TCGA-LIHC samples with survival information and excluding patients with survival time < 31 days, 344 HCC patients were finally screened. 109 genes significantly associated with prognosis were finally screened from 299 senescence-related genes (Table S3).

## WGCNA

Using the WGCNA algorithm, these genes were assigned to different modules by clustering dendrograms, and finally, we obtained three modules, among which the turquoise module consisting of 86 senescence genes showed the highest correlation and significance, therefore, we selected the senescence genes in the turquoise module for subsequent analysis (Fig. S1).

### Construction of Senescence-Related Gene Signature

**Fig. 3:**
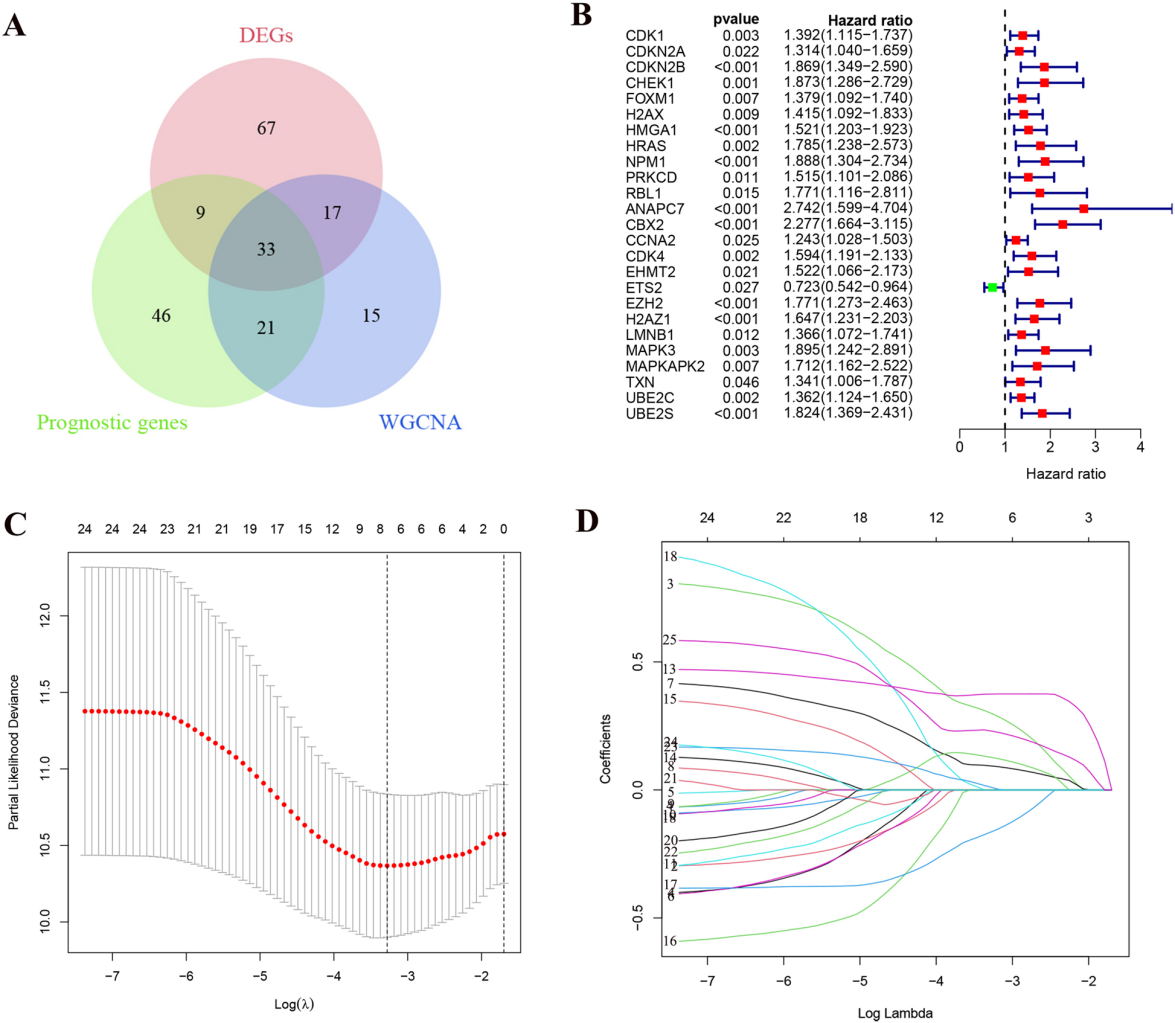
LASSO and cox regression analysis to identify signature genes in the training set. **A** We intersected the differentially expressed genes (DEGs), prognostic genes and modular genes obtained from WGCNA analysis and obtained 33 shared genes. **B** In the training set, we performed Univariate cox regression analysis on these 33 shared genes to screen for prognosis-related genes. **C** Cross-validation of the LASSO regression. **D** Coefficient value of prognostic genes

First, we took the intersection of differential expressed genes (DEGs), prognostic genes and turquoise module genes and obtained 33 intersecting genes. Using these 33 genes to construct a signature of senescence-related genes. First, 342 samples were randomly divided 1:1 into training set and Test set. These 33 intersecting genes were analyzed by univariate Cox regression to derive genes with prognostic features in the training set for the next step of analysis. Then, the signature genes and their regression coefficients were obtained by Lasso and multivariate cox regression analysis (Fig. 3A-D). Finally, we obtained 5 signature genes: CBX2, CDKN2B, ETS2, HMGA1 and UBE2S. Therefore, we created a risk score system based on the signature genes and regression coefficients to calculate the risk score for each sample. In each data set, the sample was divided into high-risk and low-risk groups based on the median value of the risk scores. The risk score is calculated as follows:

$$\mathrm{RiskScore}= (\mathrm{CBX}2*0.385) + (\mathrm{CDKN}2\mathrm{B}*0.417) + (\mathrm{ETS}2*-0.322) + (\mathrm{HMGA}1*0.197) + (\mathrm{UBE}2\mathrm{S}*0.301)$$

**Fig. 4:**
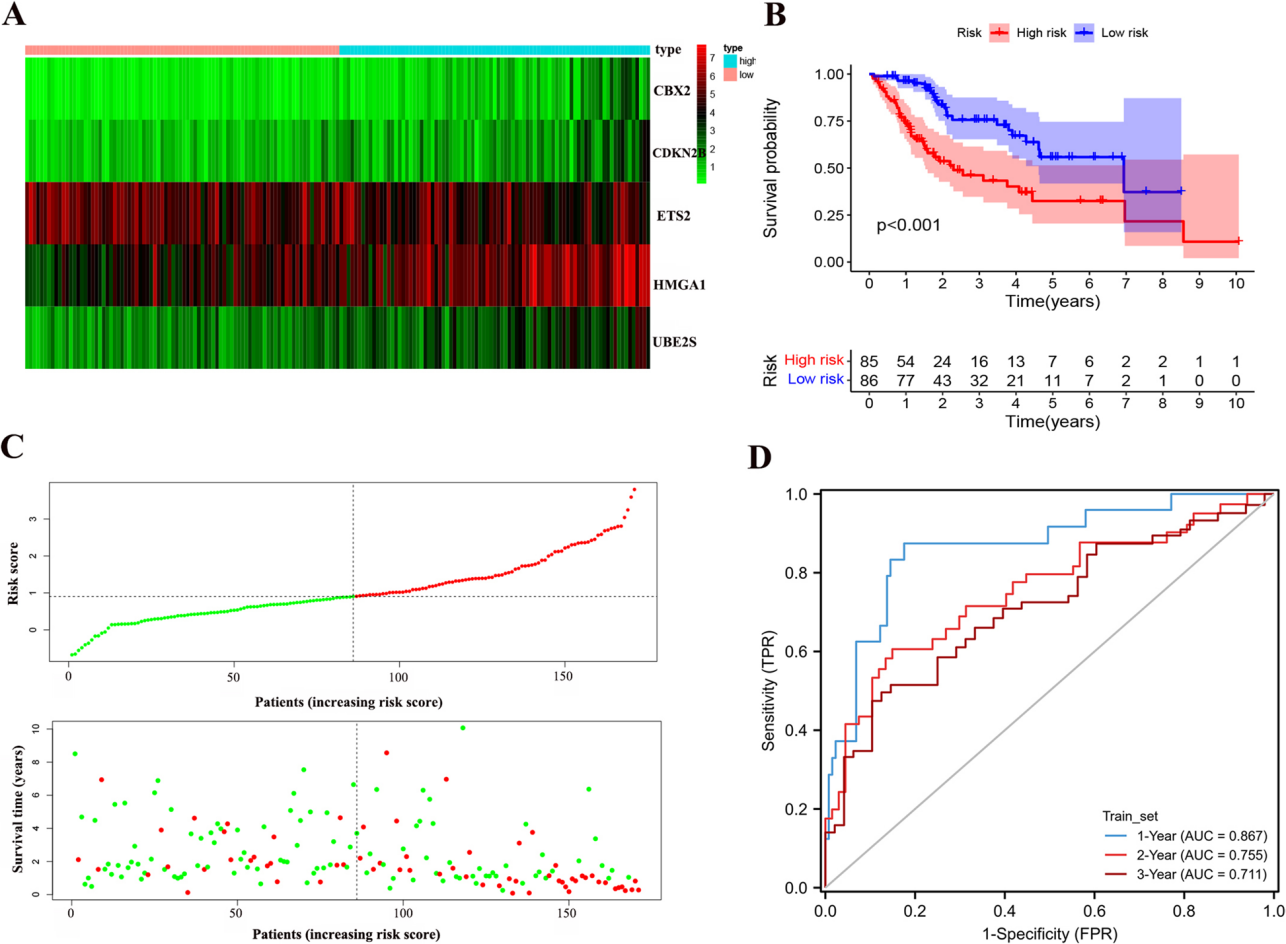
Prognostic signature based on 5 senescence-related genes. **A** The expression of 5 senescence-related genes in the TCGA train set. **B** Survival analysis between the high-risk and low-risk groups in the TCGA train set. **C** Distribution of risk scores and survival outcomes. **D** Receiver operating characteristic curve (ROC) of risk score in the TCGA train set

The expression of signature genes was demonstrated with a heat map (set 1) (Fig. 4A), and by analysis it was found that patients in the high-risk group had a lower survival rate (*p* < 0.05) (Fig. 4B). As the risk score gets higher, the survival time gets shorter and the number of deaths gets higher (Fig. 4C). The AUC values at 1, 2 and 3 years were 0.867, 0.755 and 0.711, respectively (Fig. 4D).

### Expression Validation of Signature Genes

**Fig. 5:**
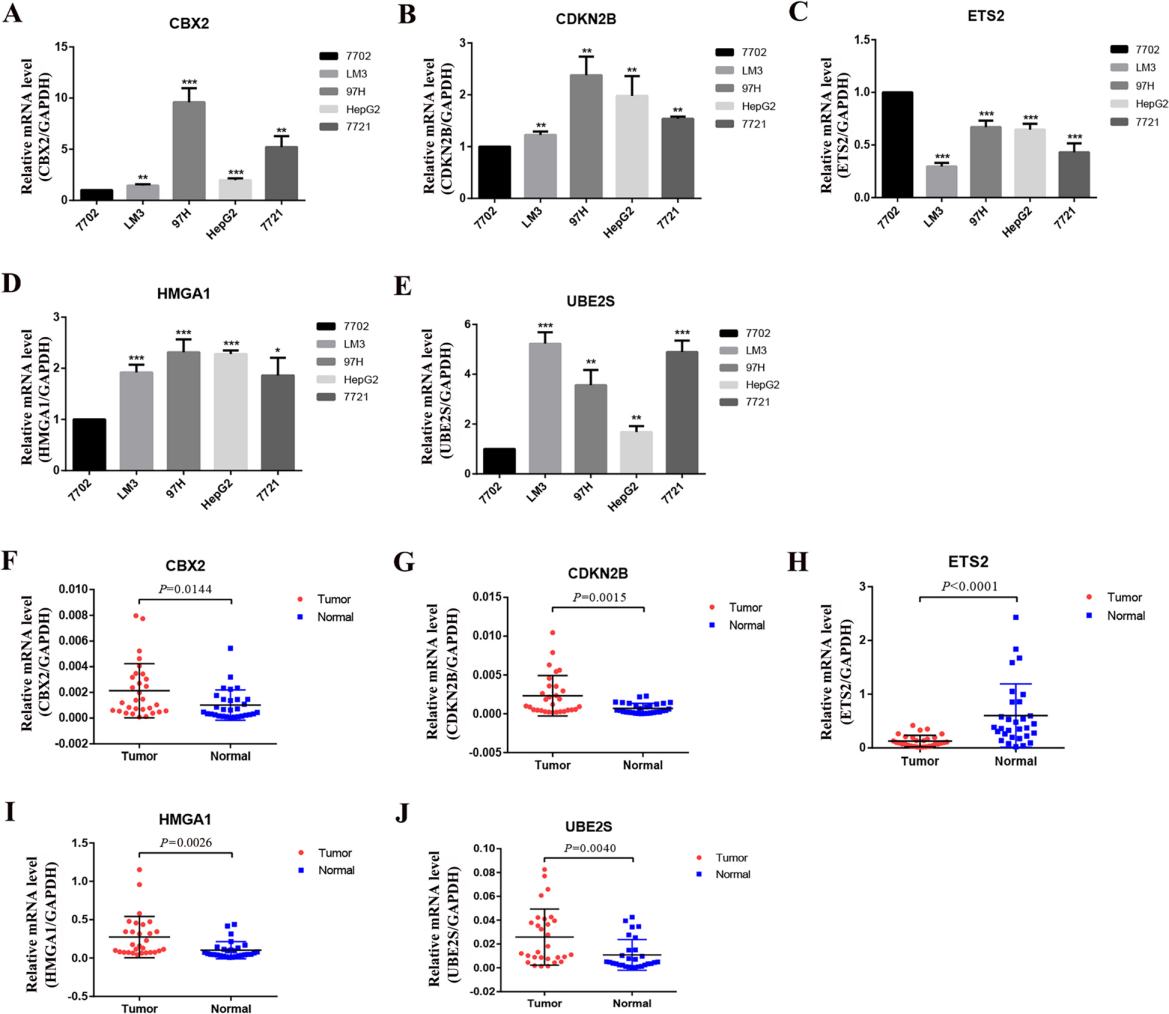
Validation of differential expression of 5 signature genes in cells and tissues by q-PCR. **A**-**E** Differential mRNA expression of 5 signature genes in 7702, LM3, 97H, HepG2 and 7721 cells. The results showed that the expression of CBX2, CDKN2B, HMGA1 and UBE2S in HCC cells was significantly higher than that in normal liver cells. In contrast, the expression of ETS2 was higher in normal liver cell. **F**-**J** Differential mRNA expression of 5 signature genes in HCC tissue and paraneoplastic tissue (30 pairs), The expression of CBX2, CDKN2B, HMGA1 and UBE2S were significantly higher in HCC and paraneoplastic tissues. In contrast, ETS2 was lower expressed in HCC tissues

First, the expression levels of the signature genes in cells (normal liver cells vs hepatocellular carcinoma cells) and tissues (hepatocellular carcinoma tissues vs paraneoplastic tissues) were verified by q-PCR assay (Fig. 5A-J).

**Fig. 6:**
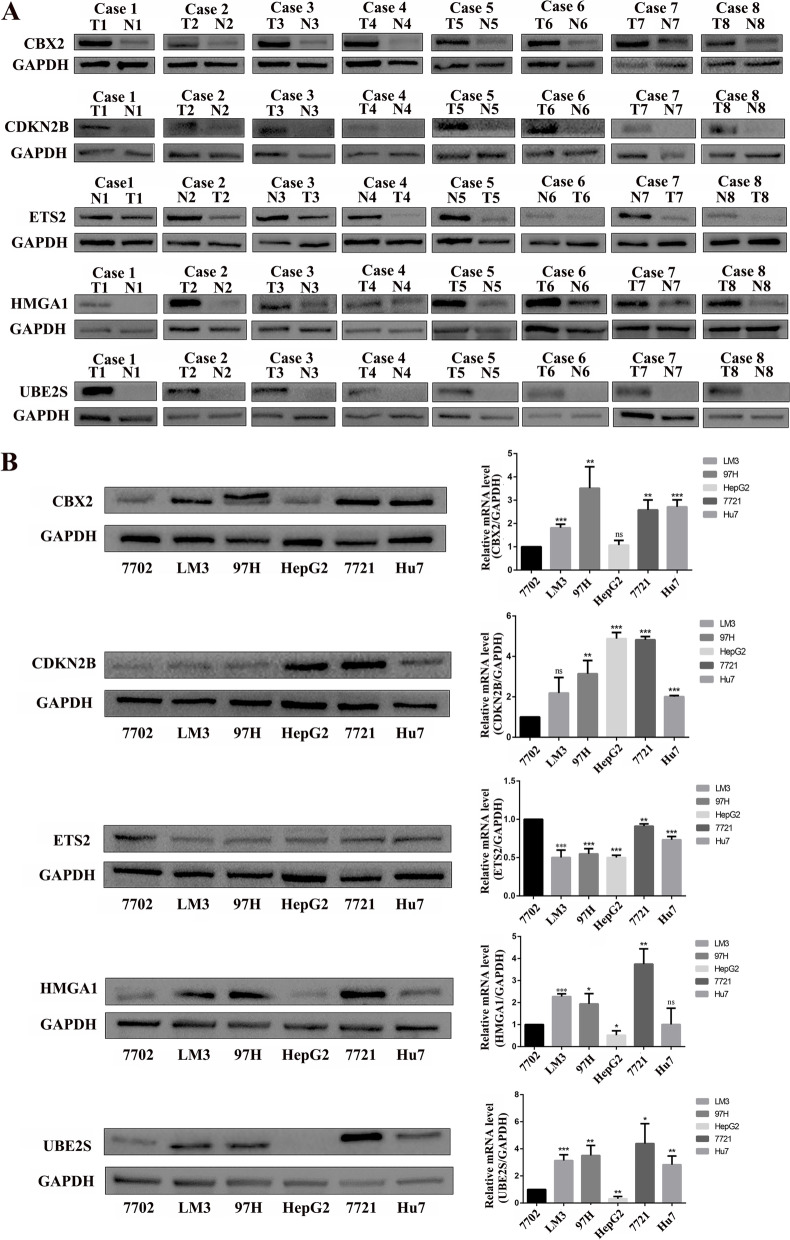
Western Blot showed the protein expression difference of 5 signature genes in HCC tissues and cells. **A** Differential protein expression of 5 signature genes in 8 pairs of HCC tissues and paraneoplastic tissues. **B** Differential protein expression of 5 signature genes in normal liver cell (7702) and HCC cells (LM3, 97H, HepG2, 7721 and Hu7)

Then, we examined the differences in expression of the signature genes in eight pairs of hepatocellular carcinoma and paraneoplastic tissues by Western blot (Fig. 6A). At the cellular level, we compared the protein expression differences of the signature genes between normal liver cell (7702) and HCC cells (LM3, 97H, HepG2, 7721 and Hu7) (Fig. 6B).

**Fig. 7:**
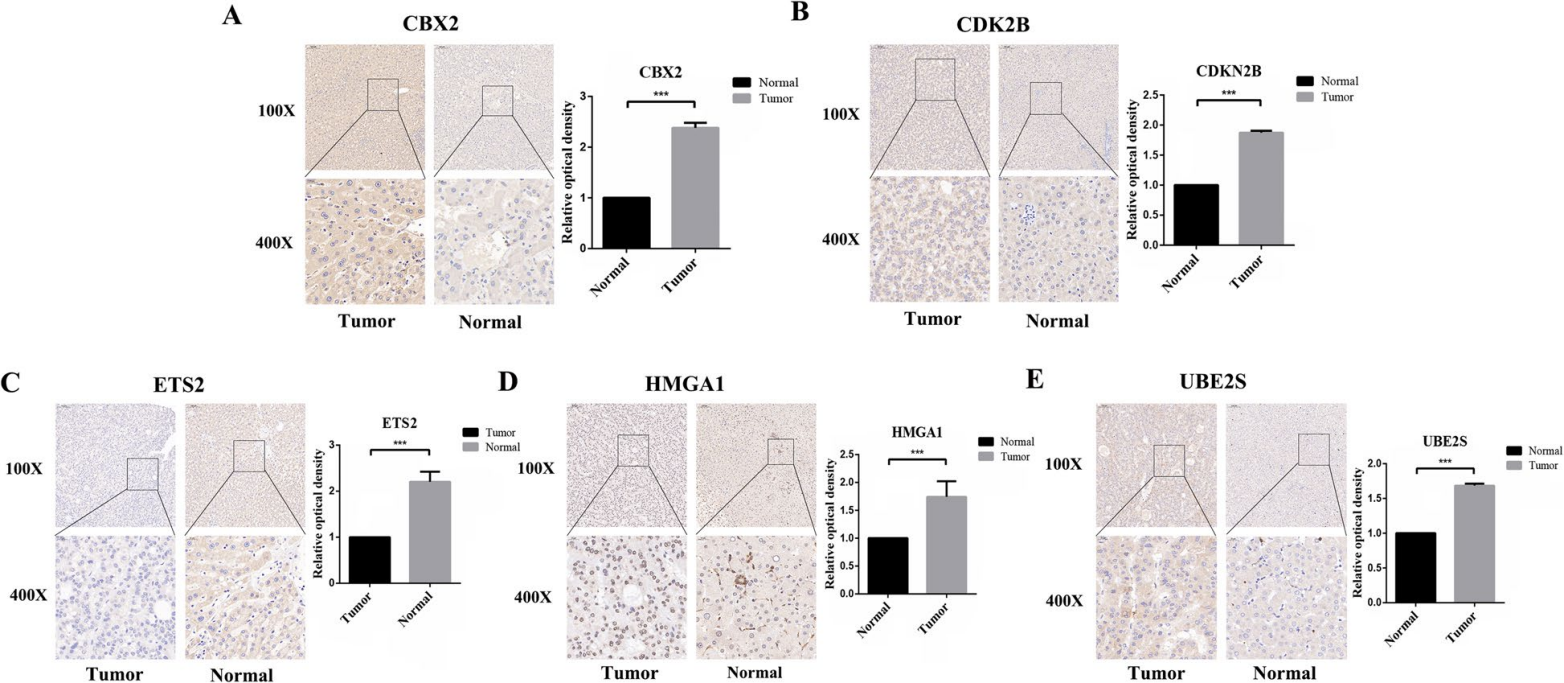
Staining images of five signature genes in HCC tissues and paraneoplastic tissues. Relative optical density scores were used to compare the differences between the two groups. **A** CBX2; **B** CDKN2B; **C** ETS2; **D** HMGA1; **E** UBE2S

Finally, we carried out an immunohistochemical experiment. We showed representative images of five genes, and then compared the expression differences between HCC tissues and paraneoplastic tissues using relative optical density scores (Fig. 7).

Molecular Interaction Networks of Signature Genes and Signature Validation.

We used the GeneMANIA (http://genemania.org/) online website to analyze the molecular interaction network between the five signature genes and found that the functions of these five genes and interacting genes (e.g. ID1, ERF, CDK4, PIAS2, CDK6,CDKN2C, etc.) were mainly related to regulation of cellular senescence, cyclin-dependent protein serine/threonine kinase regulator activity, regulation of G1/S transition of mitotic cell cycle, ubiquitin ligase complex, negative regulation of cell cycle phase transition, protein kinase inhibitor activity and nuclear ubiquitin ligase complex and CDKN2B seems to be enriched with even more features (Figure S2).

In the test group (set2), survival was worse in the high-risk group (*p* < 0.05). The AUC values at 1, 2 and 3 years were 0.745, 0.734 and 0.719, respectively (Figure S3A-C).

In the total TCGA set (set3), to validate the extent of classification between risk groups, we used t-SNE and PCA downscaling, and we found that the samples between risk groups could be well differentiated between HCC patients (Figure S4A-B). Again, the low-risk group had better survival (*p* < 0.05). The AUC values at 1, 2, and 3 years were 0.810, 0.748, and 0.719, respectively (Figure S3D-F).

The signature was next tested using ICGC data, and we also used t-distributed Stochastic Neighbor Embedding (t-SNE) and Principal Component Analysis (PCA) downscaling analysis, and found that the samples between risk groups also distinguished HCC patients well (Figure S4C-D), In addition, the survival analysis was consistent with the results of the other validation sets (Figure S3G-I).

In addition, to verify the accuracy and reliability of our signature, our signature was compared with four signatures from previous studies, and it was found that the consistency index (C-index) of our signature was higher. Meanwhile, we constructed signatures with our data using the signature genes of four other studies, and then obtained ROC curves for 1, 2, and 3 years, and found that the predictive power of our signature has higher accuracy (Figure S5).

### Independent Prognostic Analysis

**Fig. 8:**
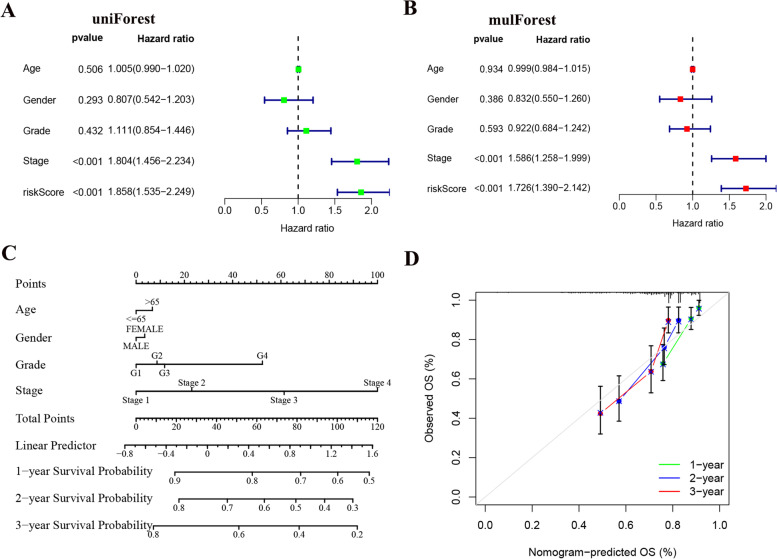
Independent prognostic analysis and Construction and validation of the Nomograms. **A** Univariate cox regression analysis for the TCGA cohort. **B** Multivariate cox regression analysis for the TCGA cohort. **C** Construction of the Nomograms. **D** The calibration curves displayed the accuracy of the nomogram in the 1-, 2- and 3 years

Both univariate and multifactorial analyses found that Stage staging and riskScore were significantly associated with patients (*P* < 0.05) (Fig. 8A, B). For further evaluation for individual patients, we simplified the statistical prediction signature using Nomograms. The calibration chart also shows good accuracy (Fig. 8C, D).

### Clinical Correlation Analysis

**Fig. 9:**
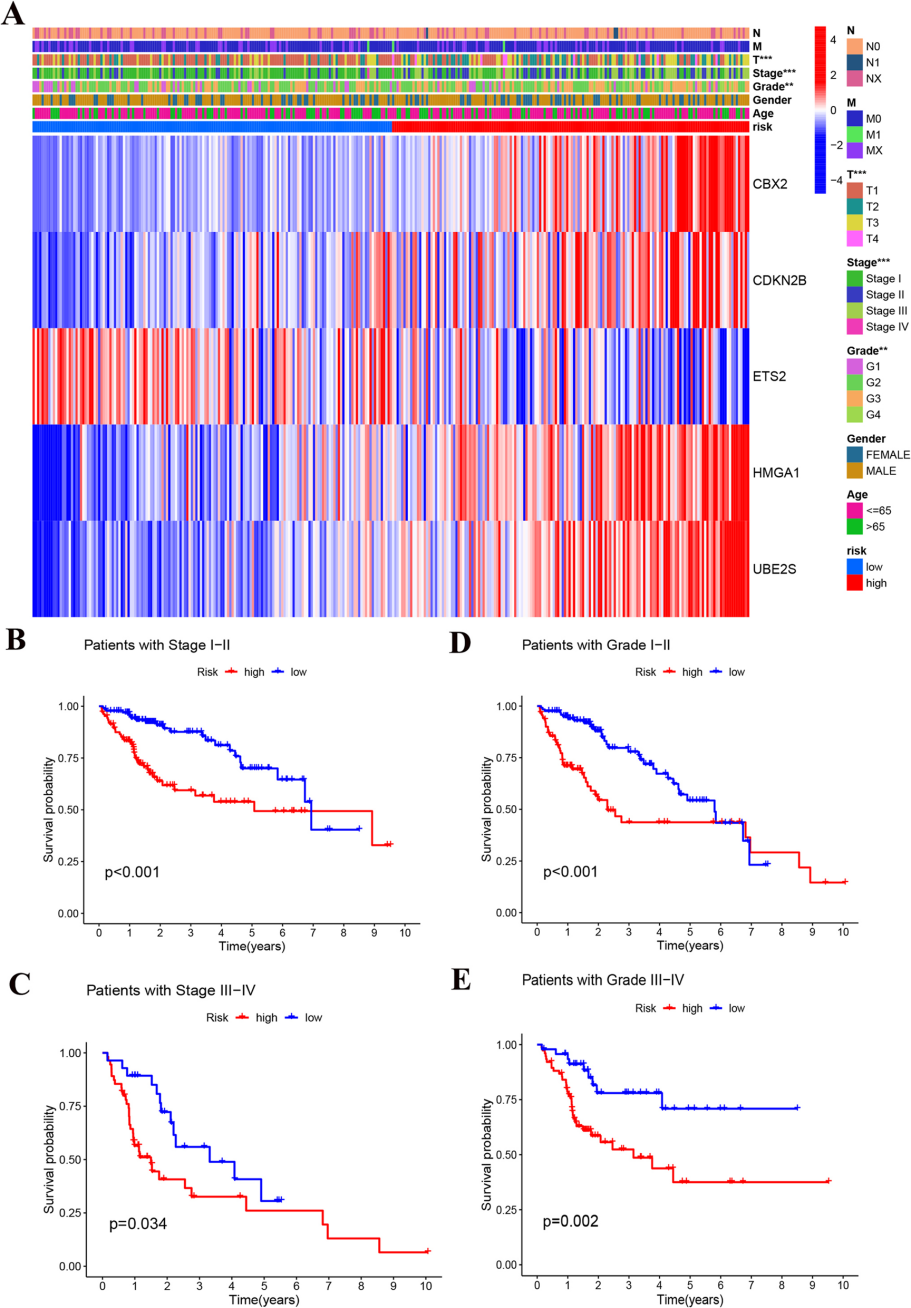
Relationship between risk score and clinical characteristics. **A** The correlations between the senescence-related genes and clinicopathologic characters of the high-risk group and low-risk group were shown as a heatmap. **B** Survival analysis between the high-risk and low-risk groups in the patients with Stage I-II. **C** Survival analysis between the high-risk and low-risk groups in the patients with Stage III-IV. **D** Survival analysis between the high-risk and low-risk groups in the patients with Grade I-II. **E** Survival analysis between the high-risk and low-risk groups in the patients with Grade III-IV. *P < 0.05, **P < 0.01, ***P < 0.001

We first presented the signature genes with clinically relevant indicators in heat map form, and found significant differences in T-stage, Stage staging and Grade staging between risk groups (*P* < 0.05) (Fig. 9A) (Figure S6). We present the clinical characteristics between risk groups in the form of box plots (Figure S7). Also, we further validated the reliability of the signature by analyzing the survival rates of patients at different clinical stages, which were low in the high-risk group in all stages and grades (*p* < 0.05) (Fig. 9B-E).

### GO and KEGG Enrichment Analysis

**Fig. 10:**
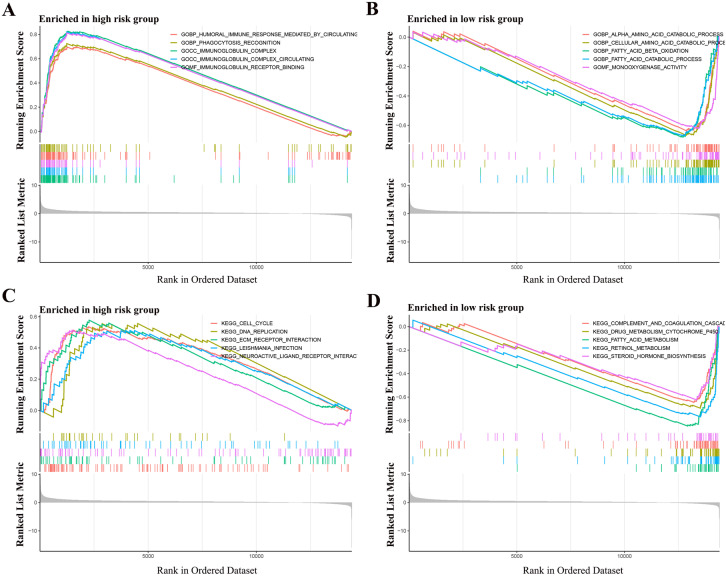
GO and KEGG enrichment analysis of senescence-related genes. **A** GO enrichment analysis of high-risk group. **B** GO enrichment analysis of low-risk group. **C** KEGG enrichment analysis of high-risk group. **D** KEGG enrichment analysis of low-risk group

GO enrichment analysis suggested that the high risk group was mainly associated with HUMORAL_IMMUNE_RESPONSE_MEDIATED_BY_CIRCULATING_IMMUNE, PHAGOCYTOSIS_RECOGNITION RECOGNITION and IMMUNOGLOBULIN_COMPLEX. The low-risk group is mainly associated with ALPHA_AMINO_ACID_CATABOLIC_PROCESS, CELLULAR_AMINO_ACID_CATABOLIC_PROCESS and FATTY_ACID_BETA_OXIDATION (Fig. 10A,B).

KEGG enrichment analysis suggested that the high-risk group was mainly associated with CELL_CYCLE, DNA_REPLICATION and ECM_RECEPTOR_INTERACTION. The low-risk group is mainly associated with COMPLEMENT_AND_COAGULATION_CASCADES, DRUG_METABOLISM_CYTOCHROME_P450 and FATTY_ACID_METABOLISM (Fig. 10C,D).

### Immune Cell Infiltration Analysis

**Fig. 11:**
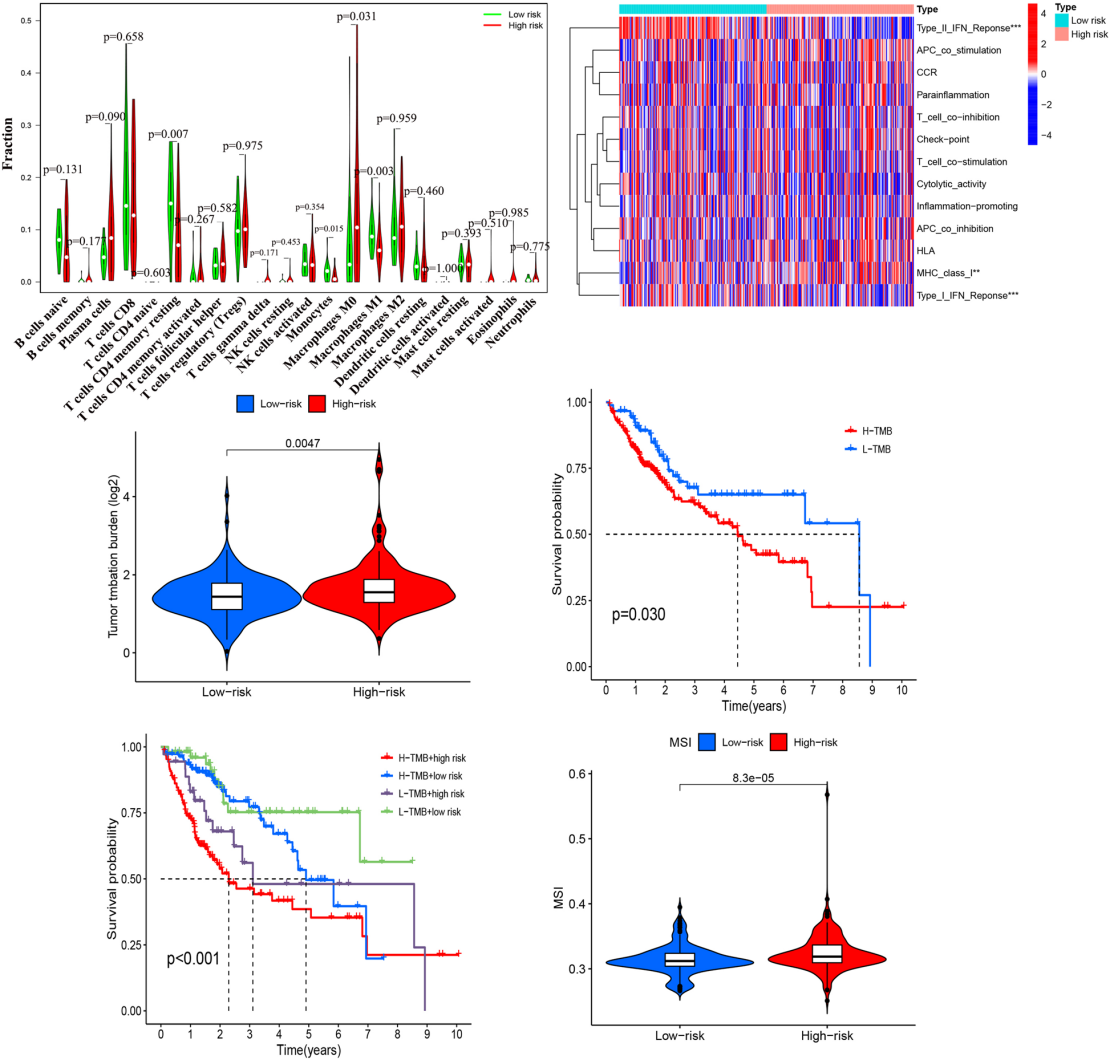
Immune infiltration analysis and Analysis of the tumor mutation burden (TMB) and Microsatellite instability (MSI). **A** The comparison of CIBERSORT scores derived from 22 different immune cells. **B** Analysis of immune-related pathways between high-risk group and low-risk group. **C** The expression of TMB between high-risk group and low-risk group. **D** Survival analysis between the high-TMB and low-TMB groups. **E** Survival analysis between the risk score and TMB levels. **F** The expression of MSI between high-risk group and low-risk group

In the high-risk group, patients had higher levels of T-cell follicular helpers, T-cell regulation (Tregs), T-cell CD4 memory activation, and macrophage M0, B-cell memory (*P* < 0.05). However, the level of resting T-cell CD4 memory, monocytes, macrophage M1 and mast cells were lower (*P* < 0.05) (Table S4) (Fig. 11A). Also, Analysis of immune-related functions between the risk groups revealed significant differences in type II_IFN_response, MHC_class_I and type I_IFN_response between the two groups (Fig. 11B). We also show immune cells that have significant correlation with signature genes (Figure S8).

### Tumor Microenvironment Analysis

Tumor microenvironment analysis showed significant differences in TMB levels between risk groups, with higher risk groups having higher levels of TMB expression, while higher TMB levels were associated with lower survival rates (*P* < 0.05) (Fig. 11C, D). The TMB levels between different risk groups affected the survival rate of patients (*P* < 0.05) (Fig. 11E). Microsatellite instability analysis showed lower MSI levels in the low-risk group (*P* < 0.05) (Fig. 11F).

### Immune Checkpoint and Drug Sensitivity Analysis

**Fig. 12:**
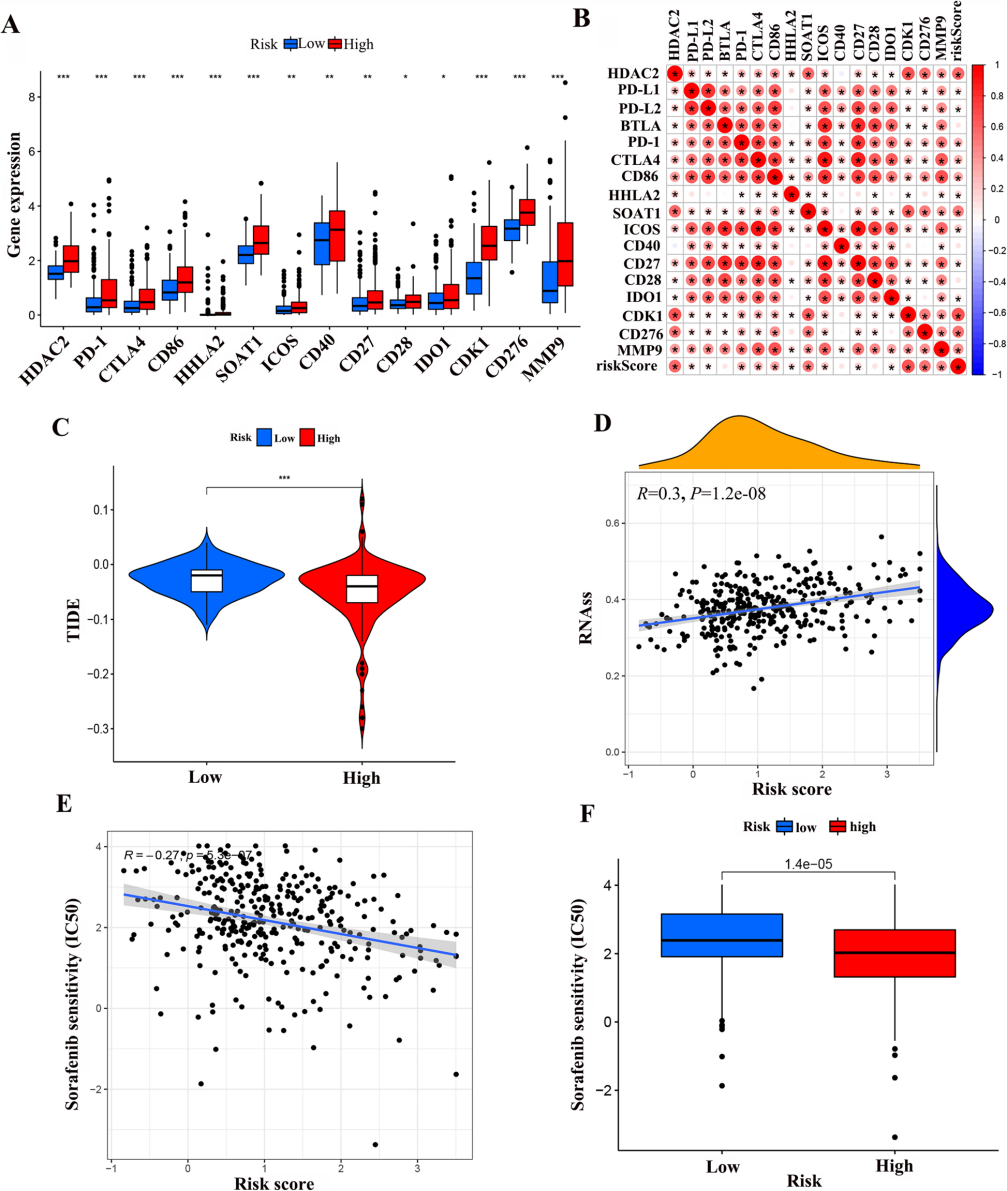
Evaluation of immune checkpoint profiles and immunotherapy between risk groups. **A** The expression of immune checkpoints between high-risk group and low-risk group. **B** Correlation analysis between immune checkpoints and risk scores. **C** Comparison of the scores of TIDE between the high and low risk group. **D** The relationship between risk score and RNAss. **E** The relationship between risk score and sorafenib senstivity (IC50). **F** Comparison of the sorafenib senstivity (IC50) between the high and low risk group

Differential analysis results showed that HDAC2, PD-1, CTLA4, CD86, HHLA2, SOAT1, ICOS, CD40, CD27, CD28, IDO1, CDK1, CD276 and MMP9 were more expressed in the high-risk group (Fig. 12A). The results of correlation analysis showed significant correlations between 14 immune checkpoints and risk scores (Fig. 12B). In addition, the high-risk group had lower TIDE scores, less likelihood of immune escape, and better efficacy during immunotherapy (Fig. 12C). In recent years, tumor progression, metastasis, recurrence and tumor resistance to cytotoxic therapy play a key role, We analyzed the correlation between risk score and tumor stem cells and found that the higher the risk score, the higher the tumor stem cell score (*P* < 0.05) (Fig. 12D).

Meanwhile, In order to analyse the differences in immunotherapy between the high and low risk groups, we used the "pRRophetic" R package to predict the gene expression and drug sensitivity of the cell lines. The analysis of riskScore and drug sensitivity showed that the higher the risk score, the higher the sensitivity to sorafenib and the better the treatment outcome is likely to be (Fig. 12E). And the high-risk group was more sensitive to sorafenib treatment (Fig. 12F).

## Discussion

In this study, we established the first signature constructed from cellular senescence genes in hepatocellular carcinoma, which provides promising new molecular markers and predictors of immunotherapy and chemotherapy through the study of cellular senescence and provides new insights for individualized treatment of hepatocellular carcinoma.

Senescent cells are characterized by persistent growth arrest and activation of damage-sensing signaling pathways, resulting in the expression of a large number of senescence-related substances [20]. However, "quiescence" and "terminal differentiation" are also responsible for cell cycle arrest, and we want to distinguish growth arrest from cellular senescence. The active hypophosphorylated RB family is also responsible for growth arrest, but they differ greatly in the timing and mechanism of arrest [20, 21].

Tumor suppression by cellular senescence is one of the most widely known cell-intrinsic mechanisms to prevent tumor transformation [22]. Mice with major defects in apoptosis are not significantly susceptible to tumors, whereas even subtle disturbances in senescence mechanisms significantly affect cancer susceptibility [23]. And mice that lose a single copy of Trp53 or p16INK4a are susceptible to tumors [24]. Therefore, it is crucial to use the cellular senescence process to control tumor development.

In our present study, a signature constructed from senescence-related genes could accurately determine the prognosis of patients with HCC. And our experiments have confirmed that these five signature genes are significantly differentially expressed in HCC tissues and normal liver tissues. CDKN2B has been shown to be associated with the development of colorectal and gastric cancers [25, 26]. Recent studies on mouse intestinal stem cells (ISC) have shown that HMGA1 is capable of maintaining Wnt and other pathways and that HMGA1 overexpression promotes tumor development [27]. It has also been shown that HMGA1 can directly interact with PD-L1, and upregulation of HMGA1 by PD-L1 can activate PI3K/Akt and MEK/ERK pathways to promote the development of colon and intestinal cancers [28]. The polymorphic histone CBX2 plays an important role in processes involved in cell proliferation and differentiation, and its targeted deletion leads to homogeneous heteromorphic transformation, proliferation defects and premature senescence. Deletion of CBX2 in mouse fibroblasts leads to large-scale chromatin structural abnormalities and chromosomal instability [29]. Moreover, knockdown of CBX2 has previously been shown to inhibit the development of HCC [30]. ETS2 can affect the progression of osteosarcoma and gastric cancer [31, 32]. In addition, knockdown of UBE2S can inhibit the proliferation and invasion of HCC [33].

We showed significantly higher expression of the common 14 immune checkpoints and a better response to immune checkpoint inhibitors in the high-risk group. Targeted therapy is now crucial in the treatment of HCC, and sorafenib is an effective first-line therapy in advanced HCC [34]. The present study showed that senescence-related gene signatures can be used to predict treatment response to sorafenib. High-risk patients may be more sensitive to sorafenib.

## Conclusion

In summary, the senescence-related gene signature can well predict the prognosis of HCC patients, and the signature provides a new idea to improve the immunotherapy of hepatocellular carcinoma.

## Supplementary Information

**Additional file 1: Figure S1.** Weighted gene coexpression network analysis.

**Additional file 2: Figure S2.** Molecular interaction networks of signature genes.

**Additional file 3: Figure S3.** Riskscore validation.

**Additional file 4: Figure S4. **t-SNE and PCA between the high- and low-risk groups in TCGA and ICGC.

**Additional file 5: Figure S5.** Comparison with other signatures.

**Additional file 6: Figure S6.** The differences of Grade, Stage and T stages between high-risk group and low-risk group.

**Additional file 7: Figure S7.** Relationship between risk scores and clinical characteristics.

**Additional file 8: Figure S8.** Correlation between immune cells and 5 senescence-related genes.

**Additional file 9: Table S1.** Senescence-Related Genes in three gene sets.

**Additional file 10: Table S2.** Difference analysis between normal and tumor groups of LIHC.

**Additional file 11: Table S3.** Univariate cox regression for senescence-related genes.

**Additional file 12: Table S4.** The difference of 22 kinds of immune cells between high and low risk groups.

## Data Availability

The dataset supporting the conclusions of this article is included within the article and its additional file. TCGA-LIHC is available at (https://portal.gdc.cancer.gov/); ICGC is available at (https://dcc.icgc.org/). HPA is available at (https://www.proteinatlas.org/).
