# Supplementary material for: Comprehensive Analysis of Cellular Senescence-Related Genes in Prognosis, Molecular Characterization and Immunotherapy of Hepatocellular Carcinoma
Source: Biol Proced Online. 2022 Dec 19;24:24. doi: 10.1186/s12575-022-00187-7 (PMC9761989; doi:10.1186/s12575-022-00187-7)
Supplement: Supplementary file 1 — Additional file 1: Figure S1. Weighted gene coexpression network analysis. [file 12575_2022_187_MOESM1_ESM.docx]

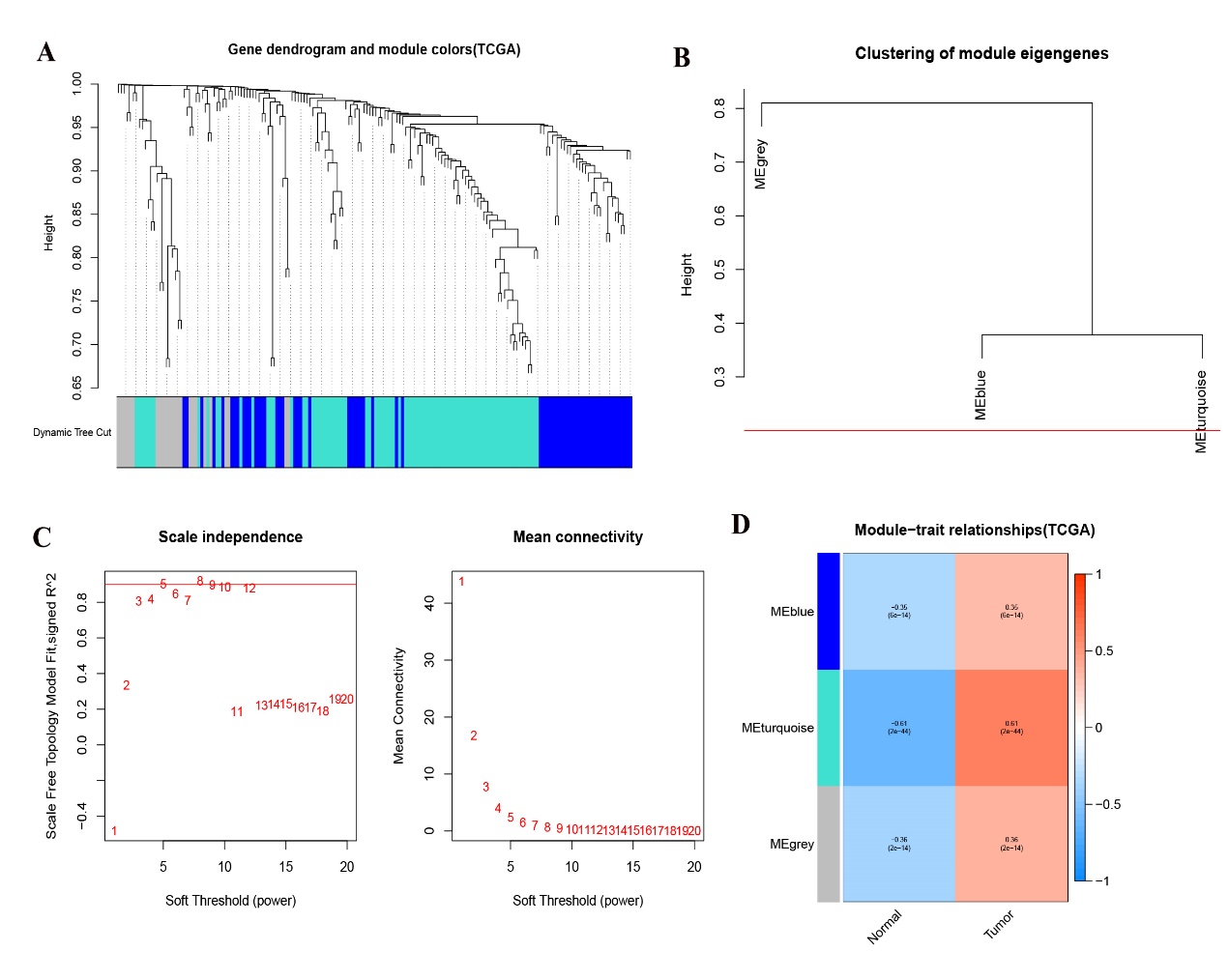


**FIGURE S1 | Weighted gene coexpression network analysis. (A)** Clustering dendrogram of genes, with dissimilarity based on topological overlap, together with assigned module colors. **(B)** Visualization of the eigengene network representing the relationships among the modules and the clinical trait weight. **(C)** Analysis of network topology for various soft-thresholding powers. **(D)** Module-trait associations: Each row corresponds to a module eigengene and the column to the tumor and normal. Each cell contains the corresponding correlation and p-value.
