# Supplementary figures and images for: Comprehensive Analysis of Cellular Senescence-Related Genes in Prognosis, Molecular Characterization and Immunotherapy of Hepatocellular Carcinoma
Source: Biol Proced Online. 2022 Dec 19;24:24. doi: 10.1186/s12575-022-00187-7 (PMC9761989; doi:10.1186/s12575-022-00187-7)

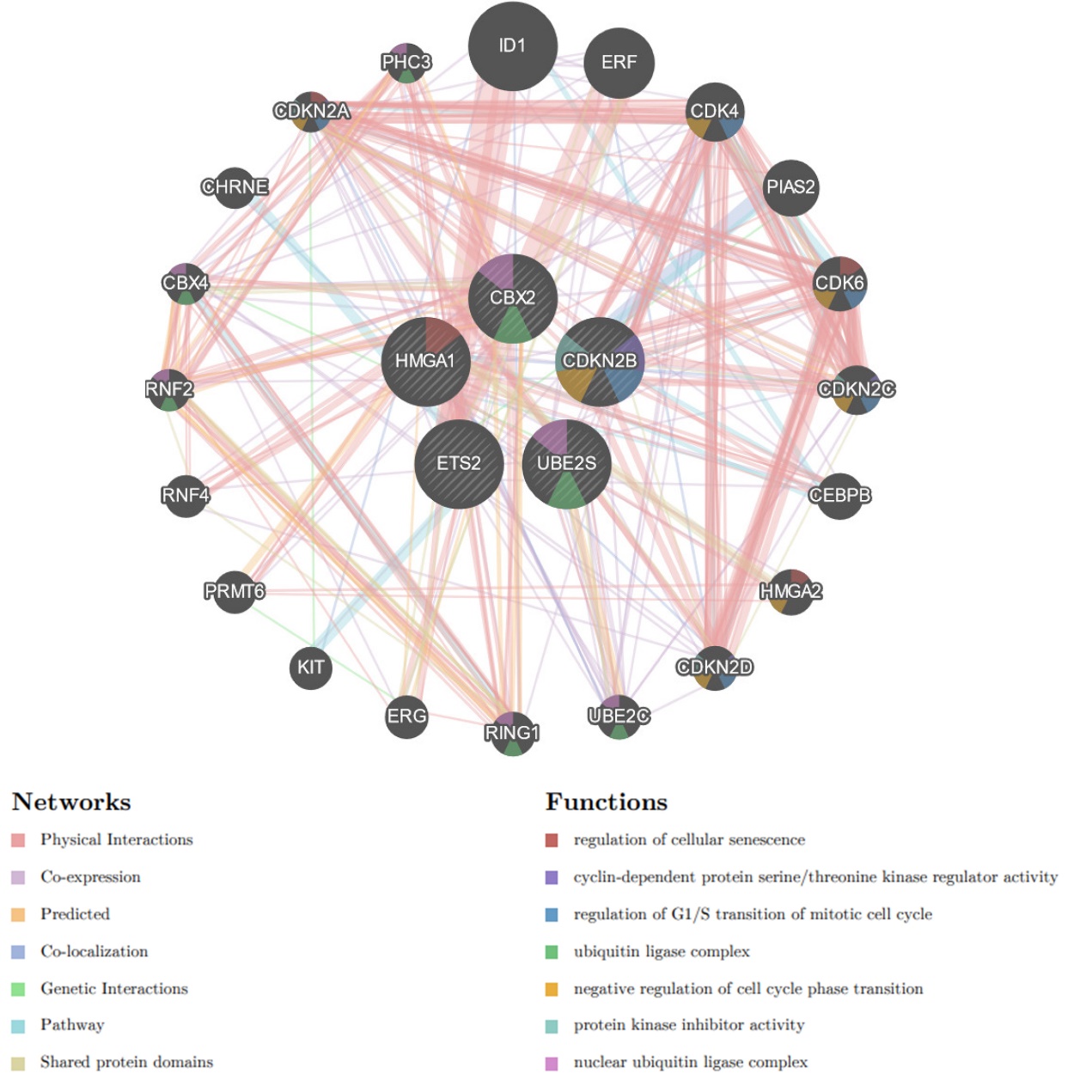


**FIGURE S2 | Molecular interaction networks of signature genes.**

Supplement: Supplementary file 2 — Additional file 2: Figure S2. Molecular interaction networks of signature genes. [file 12575_2022_187_MOESM2_ESM.docx]

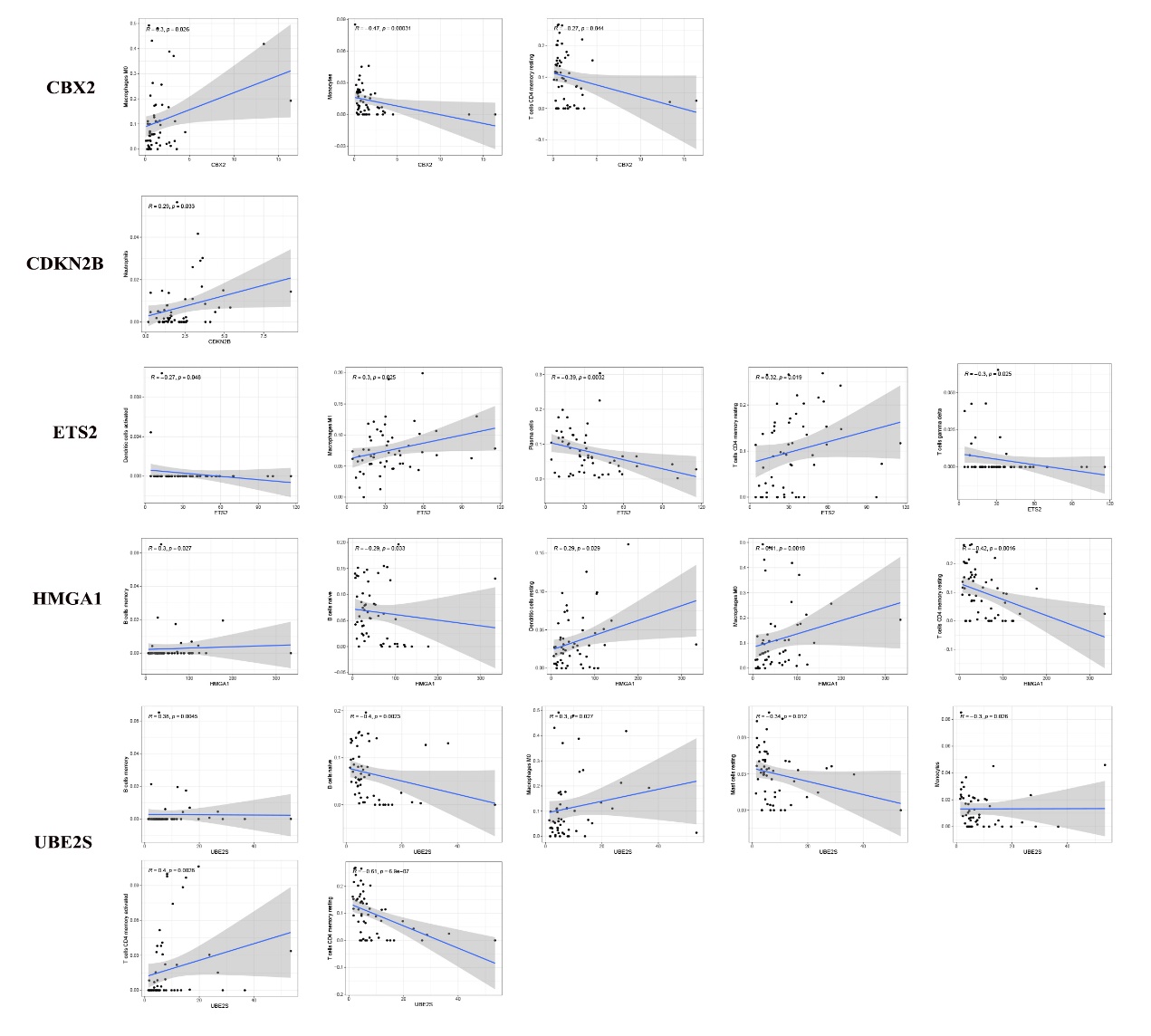


**FIGURE S8 |** Correlation between immune cells and 5 senescence-related genes.

Supplement: Supplementary file 8 — Additional file 8: Figure S8. Correlation between immune cells and 5 senescence-related genes. [file 12575_2022_187_MOESM8_ESM.docx]
