# Supplementary material for: Comprehensive Analysis of Cellular Senescence-Related Genes in Prognosis, Molecular Characterization and Immunotherapy of Hepatocellular Carcinoma
Source: Biol Proced Online. 2022 Dec 19;24:24. doi: 10.1186/s12575-022-00187-7 (PMC9761989; doi:10.1186/s12575-022-00187-7)
Supplement: Supplementary file 3 — Additional file 3: Figure S3. Riskscore validation. [file 12575_2022_187_MOESM3_ESM.docx]

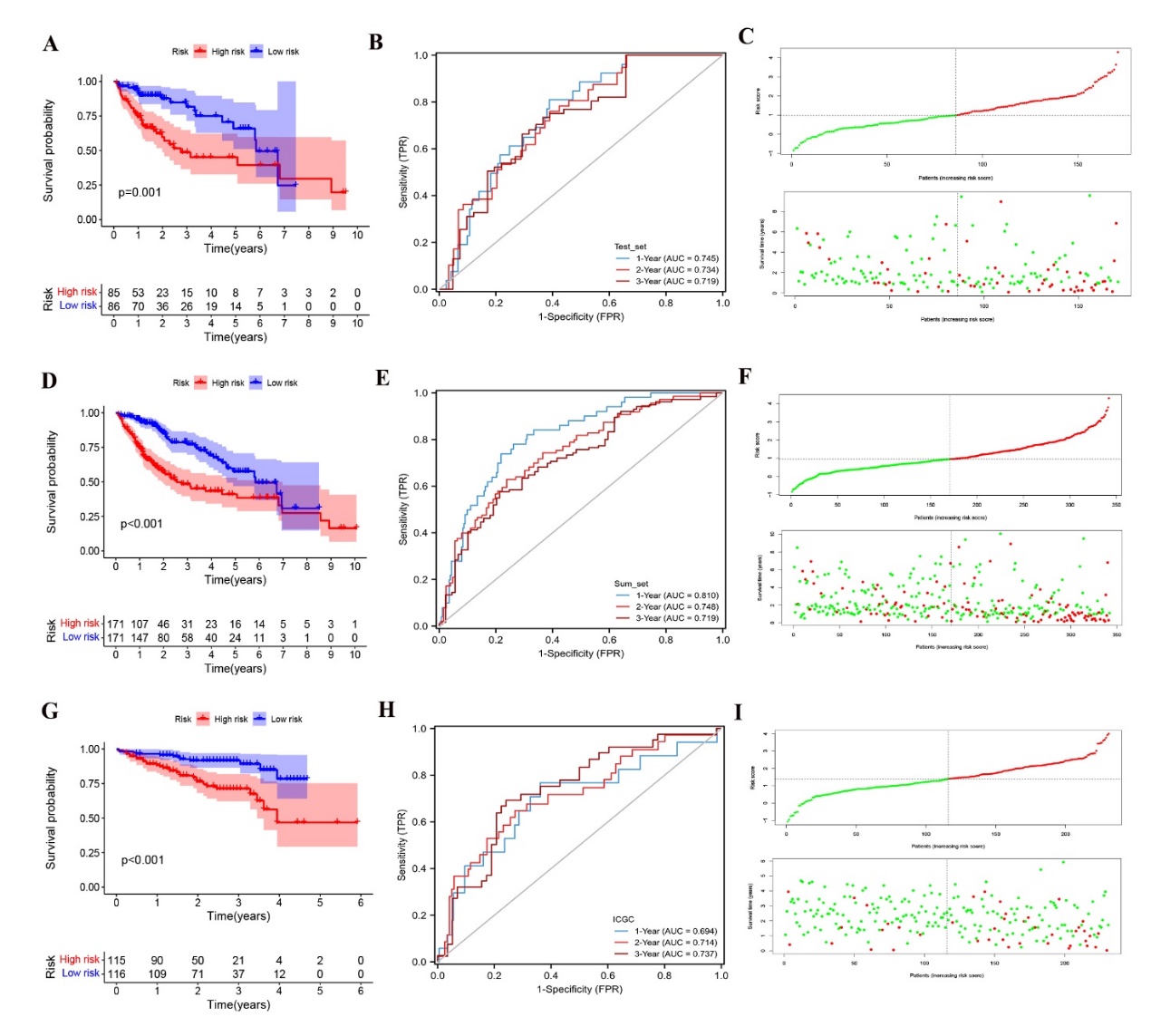


**FIGURE S3 | Riskscore validation.** Survival analysis between the high-risk and low-risk groups in the TCGA test set**(A)**, sum set**(D)** and ICGC set**(G)**. Receiver operating characteristic curve(ROC) of risk score in the TCGA test set**(B)**, sum set**(E)** and ICGC set**(H)**. Distribution of risk scores and survival outcomes in the TCGA test set**(C)**, sum set**(F)** and ICGC set**(I)**.
