# Supplementary material for: Comprehensive Analysis of Cellular Senescence-Related Genes in Prognosis, Molecular Characterization and Immunotherapy of Hepatocellular Carcinoma
Source: Biol Proced Online. 2022 Dec 19;24:24. doi: 10.1186/s12575-022-00187-7 (PMC9761989; doi:10.1186/s12575-022-00187-7)
Supplement: Supplementary file 4 — Additional file 4: Figure S4. t-SNE and PCA between the high- and low-risk groups in TCGA and ICGC. [file 12575_2022_187_MOESM4_ESM.docx]

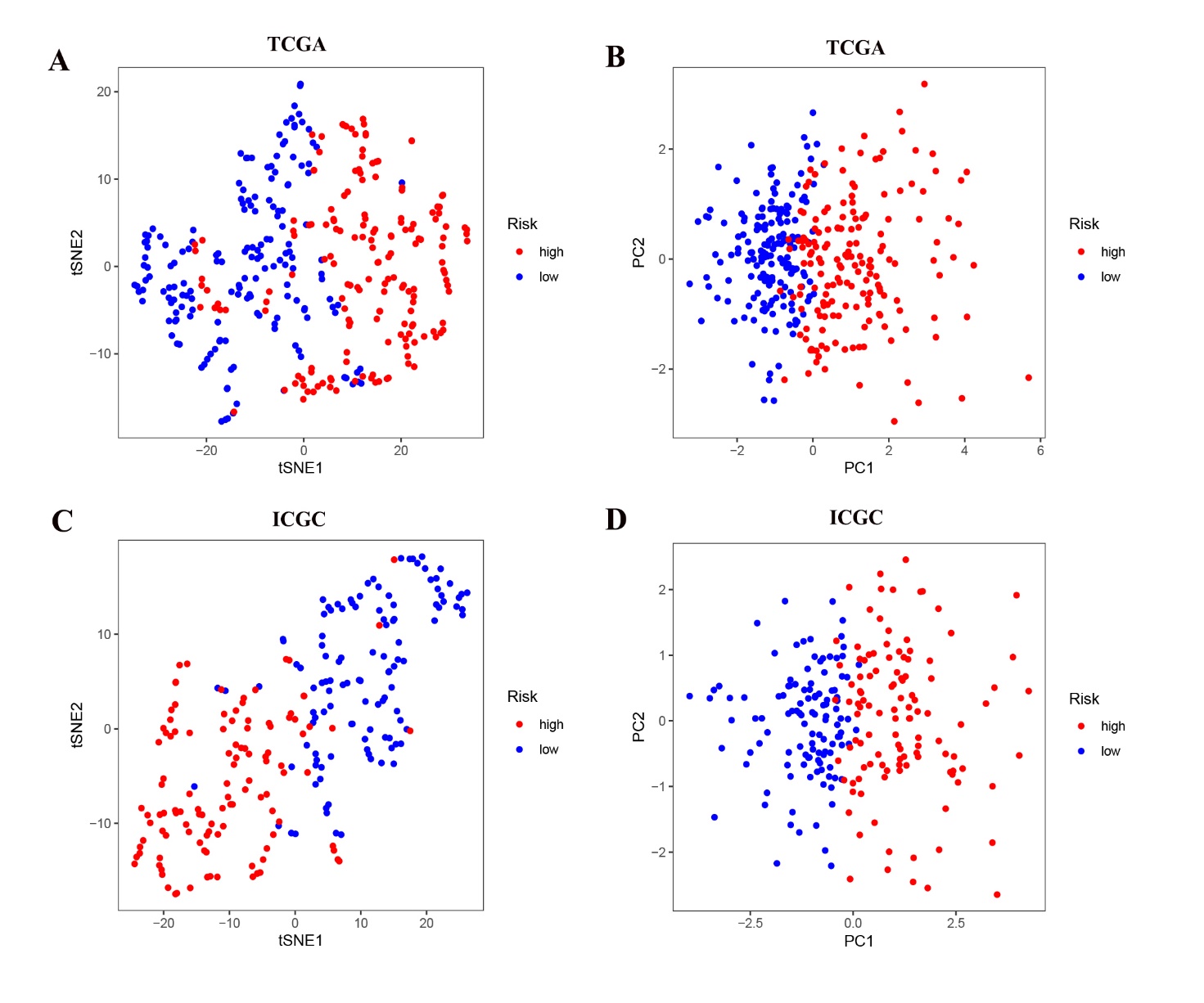


**FIGURE S4 | t-SNE and PCA between the high- and low-risk groups in TCGA and ICGC. (A)** t-SNE analysis in TCGA. **(B)** PCA in TCGA. **(C)** t-SNE analysis in ICGC. **(D)** PCA in ICGC.
