# Supplementary material for: Comprehensive Analysis of Cellular Senescence-Related Genes in Prognosis, Molecular Characterization and Immunotherapy of Hepatocellular Carcinoma
Source: Biol Proced Online. 2022 Dec 19;24:24. doi: 10.1186/s12575-022-00187-7 (PMC9761989; doi:10.1186/s12575-022-00187-7)
Supplement: Supplementary file 5 — Additional file 5: Figure S5. Comparison with other signatures. [file 12575_2022_187_MOESM5_ESM.docx]

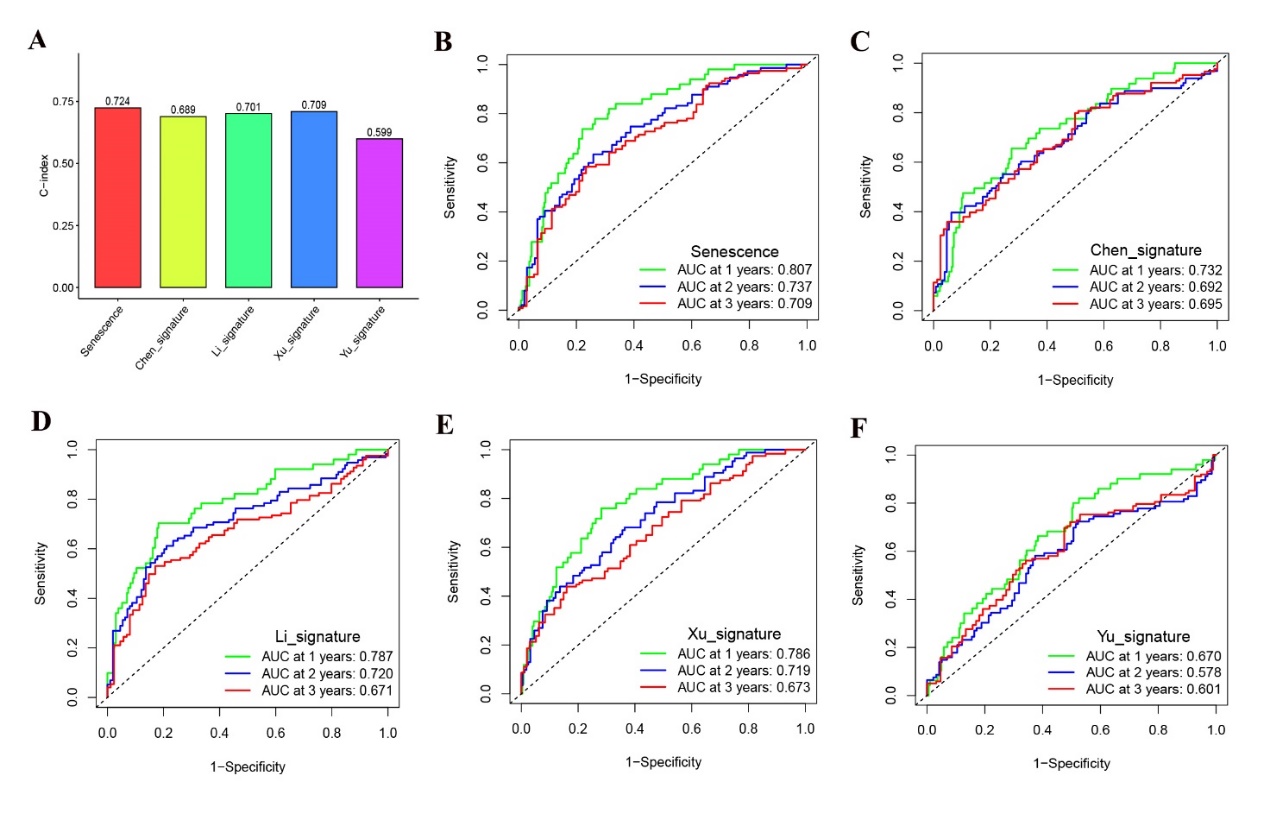


**FIGURE S5 | Comparison with other signatures. (A)** We compared the concordance index (C-index) of our signature with the hepatocellular carcinoma signature from previous four studies to determine the accuracy of our signature. **(B)** The ROC curve of our senescence signature. **(C)** The ROC curve of Chen_signature. **(D)** The ROC curve of Li_signature. **(E)** The ROC curve of Xu_signature. **(F)** The ROC curve of Yu_signature.
