# Supplementary material for: Comprehensive Analysis of Cellular Senescence-Related Genes in Prognosis, Molecular Characterization and Immunotherapy of Hepatocellular Carcinoma
Source: Biol Proced Online. 2022 Dec 19;24:24. doi: 10.1186/s12575-022-00187-7 (PMC9761989; doi:10.1186/s12575-022-00187-7)
Supplement: Supplementary file 6 — Additional file 6: Figure S6. The differences of Grade, Stage and T stages between high-risk group and low-risk group. [file 12575_2022_187_MOESM6_ESM.docx]

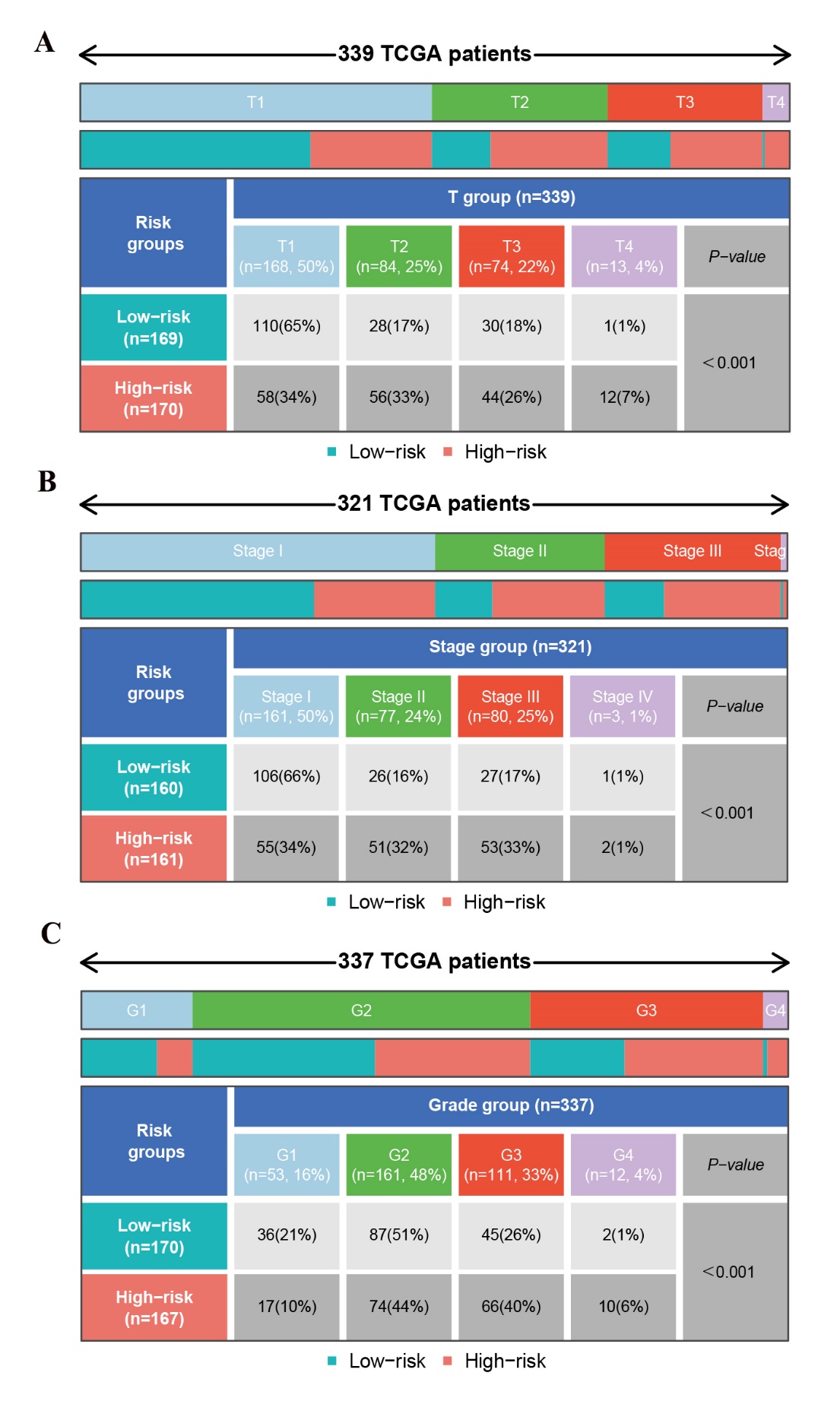


**FIGURE S6 |** **The differences of Grade, Stage and T stages between high-risk group and low-risk group.** **(A)** T stage. **(B)** Stage. **(C)** Grade.
