# Supplementary material for: Comprehensive Analysis of Cellular Senescence-Related Genes in Prognosis, Molecular Characterization and Immunotherapy of Hepatocellular Carcinoma
Source: Biol Proced Online. 2022 Dec 19;24:24. doi: 10.1186/s12575-022-00187-7 (PMC9761989; doi:10.1186/s12575-022-00187-7)
Supplement: Supplementary file 7 — Additional file 7: Figure S7. Relationship between risk scores and clinical characteristics. [file 12575_2022_187_MOESM7_ESM.docx]

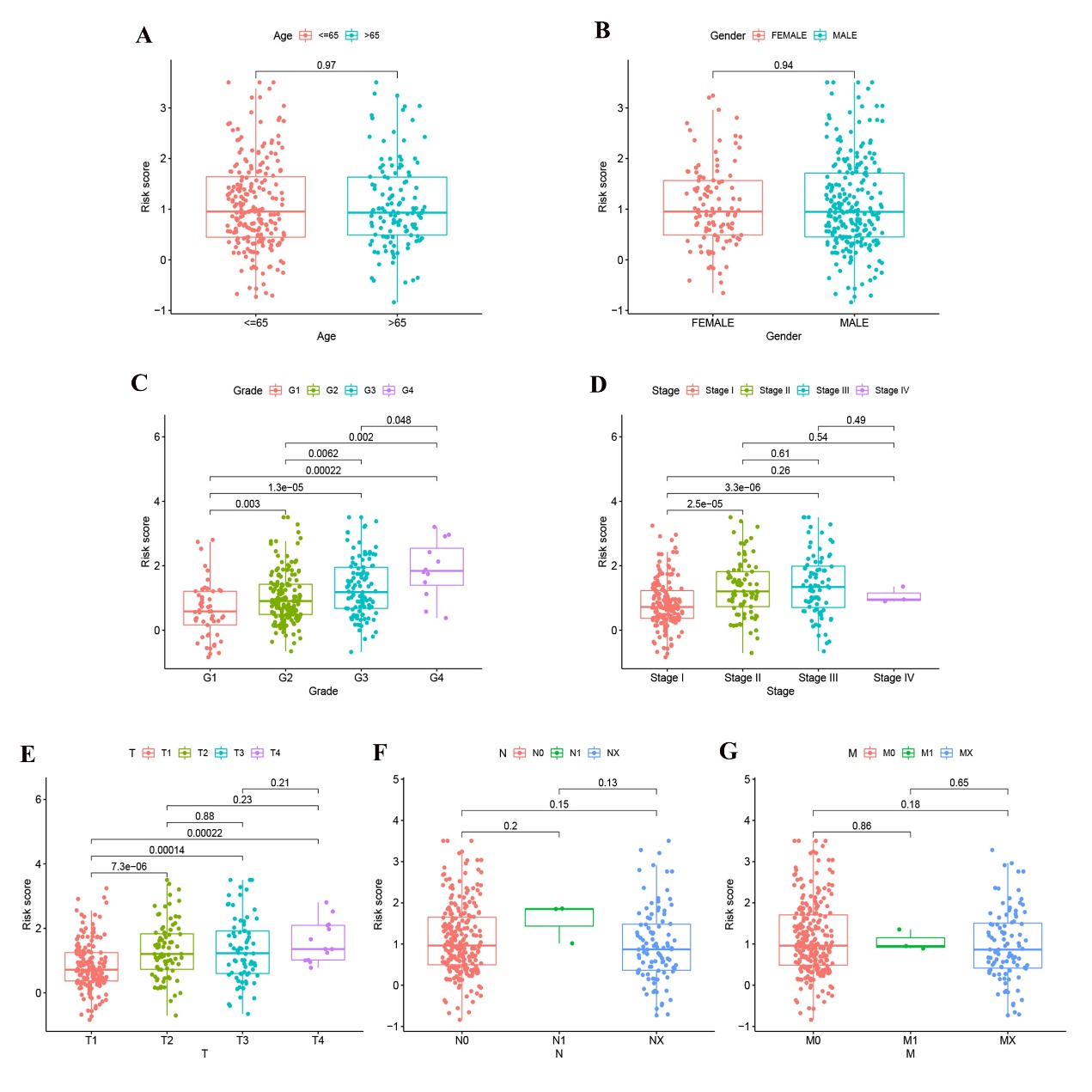


**FIGURE S7 |** Relationship between risk scores and clinical characteristics. **(A)** Age. **(B)** Gender. **(C)** Grade. **(D)** Stage. **(E)** T. **(F)** N. **(G)** M.
